# A Clustered Randomized Controlled Trial of Low Intensity Cognitive Behavioral Therapy Adapted for Carers’ Needs

**DOI:** 10.1093/geroni/igaf122.033

**Published:** 2025-12-31

**Authors:** Wai Sze Chan, Tom Chun Wai Tsoi, Yu Hon Kenneth Kwok, Vera Mun Yu Tang, Tarani Chandola, Jianchao Quan, Vivian Weiqun Lou

**Affiliations:** The University of Hong Kong, Hong Kong, China (People’s Republic); The University of Hong Kong, Hong Kong, China (People’s Republic); The University of Hong Kong, Hong Kong, China (People’s Republic); The University of Hong Kong, Hong Kong, China (People’s Republic); The University of Hong Kong, Hong Kong, China (People’s Republic); The University of Hong Kong, Hong Kong, China (People’s Republic); The University of Hong Kong, Hong Kong, China (People’s Republic)

## Abstract

Carers experience elevated levels of stress, distress, and deterioration in quality of life. A low-intensity cognitive behavioral therapy (CBT)-guided group intervention is developed for the specific needs of family carers of older adults. This intervention integrates key CBT elements including behavioral activation, mindfulness, and problem-solving, with addition of lifestyle modifications including sleep hygiene, relaxation, and healthy dietary recommendations. A two-armed, multi-site, single-blind clustered randomized controlled trial is being conducted to examine the effectiveness of the intervention. Participants are adults aged 18+ who identify as a carers of a family member aged 60+, provide caregiving for 6+ hours per week, show moderate levels of need in the Carer Multidimensional Need Assessment, and are not diagnosed Alzheimer or dementia or other acute health conditions that hinder study participation and caregiving. Primary outcomes include depressive and anxiety symptoms. Secondary outcomes include carer needs, social support, quality of life, caregiver burden, and resilience. Outcomes measures are administered at baseline, post-intervention, and at 3-month follow-up. Carers in the intervention group are hypothesized to show a greater reduction in depression and anxiety symptoms and greater improvements in secondary outcomes compared to those in the control group. The trial protocol is registered on ClinicalTrials.gov with the identifier NCT0647301. This trial seeks to provide evidence for an integrated, evidence-based intervention model that can be widely disseminated to support family carers of older adults, ultimately improving their mental health and quality of life.

